# A phase II study of capecitabine plus 3-weekly oxaliplatin as first-line therapy for patients with advanced gastric cancer

**DOI:** 10.1038/sj.bjc.6603046

**Published:** 2006-03-21

**Authors:** Y H Park, B-S Kim, B-Y Ryoo, S H Yang

**Affiliations:** 1 Department of Internal Medicine, Division of Haematology and Oncology, Seoul Veterans Hospital, Kangdong-Ku, Seoul, Korea; 2 Korea Institute of Radiological and Medical Science, Seoul Veterans Hospital, Kangdong-Ku, Seoul, Korea

**Keywords:** oxaliplatin, capecitabine, advanced gastric cancer, first-line

## Abstract

Capecitabine plus oxaliplatin every 3 weeks (XELOX regimen) has proven efficacy in patients with colorectal carcinoma. We investigated this combination in patients with previously untreated advanced gastric carcinoma. The study population comprised patients with histologically confirmed nonresectable advanced gastric adenocarcinoma. Patients received intravenous oxaliplatin 130 mg m^−2^ over 2 h on day 1 plus oral capecitabine 1000 mg m^−2^ twice daily on days 1–14, every 3 weeks. Patients received a maximum of eight cycles. Twenty evaluable patients (17 men, 3 women) with a median age of 64 years (range 38–75) were enrolled. The overall response rate was 65% (95% confidence interval (CI), 44–86%), with complete responses in two patients and partial responses in 11 patients. Median progression-free survival was 7.5 months (95% CI, 3.2–11.7 months); median overall survival was not reached during the study period. There was no grade 4 and little grade 3 toxicity. The most common haematological adverse event was anaemia (65% of patients) and the most common nonhaematological toxicities were vomiting (65%), neuropathy (60%), diarrhoea (30%), and hand–foot syndrome (20%). In conclusion, XELOX is apparently as effective as triplet combinations and is well tolerated as first-line therapy for advanced gastric carcinoma. We are starting a large multi-institutional phase II study of XELOX in this setting.

Gastric cancer remains one of the most common malignancies worldwide (Parkin *et al*, 1999), and is the leading cause of cancer-related deaths in Korea (National Statistical Office, 2001). Despite improvements in the early diagnosis of gastric cancer, many patients present with inoperable disease. Advanced gastric carcinoma remains incurable with a median survival of only 6–10 months even in patients treated with chemotherapy (Hong *et al*, 2004; Park *et al*, 2004; Thuss-Patience *et al*, 2005).

5-FU in combination with cisplatin (FP regimen) is commonly used in advanced disease because of the activity of both drugs when administered as single agents. In randomised phase III trials in advanced gastric cancer, FP led to improved response rates compared with 5-FU, doxorubicin and mitomycin (FAM) or 5-FU single-agent therapy (Kim *et al*, 1993), and showed a trend towards improved response rates compared with 5-FU, doxorubicin and methotrexate (FAMTX) or etoposide, leucovorin and bolus 5-FU (ELF) (Vanhoefer *et al*, 2000). Some European centres consider epirubicin, cisplatin and 5-FU (ECF) to be standard therapy based on its response and survival advantage over FAMTX (Waters *et al*, 1999). However, all of these 5-FU-based regimens are inconvenient to administer and have the potential for severe toxicity (i.e. renal toxicity, emesis). Therefore, more effective and better-tolerated systemic therapy is needed to improve the management of patients with advanced gastric cancer.

The oral fluoropyrimidine capecitabine (Xeloda®; F Hoffmann La-Roche) has been designed to generate 5-FU preferentially in tumours through exploitation of the significantly higher levels of thymidine phosphorylase in tumour tissue compared with healthy tissue (Ishitsuka *et al*, 1998; Schüller *et al*, 2000; Miwa *et al*, 2001). Capecitabine has shown superior response rates and relapse-free survival and superior safety compared with i.v. 5-FU/LV in the treatment of metastatic (Cassidy *et al*, 2002; Van Cutsem *et al*, 2004) and adjuvant colorectal cancer (Scheithauer *et al*, 2003a; Twelves *et al*, 2005). Capecitabine 1250 mg m^−2^ twice daily on days 1–14 every 3 weeks has also been shown to be active (overall response rate 28%; stable disease 36%) and well tolerated in a phase II study of previously untreated patients with advanced gastric cancer (Hong *et al*, 2004). A 4-weekly intermittent schedule of capecitabine (828 mg m^−2^ twice daily for 3 weeks followed by 1 week of rest) also produced overall response rates of 19 and 26% in two Japanese studies in patients with advanced gastric cancer, with a median survival of approximately 8.8 months at the time of reporting (Koizumi *et al*, 2003; Kondo *et al*, 2003).

Oxaliplatin, a chemotherapeutic agent currently being investigated in the treatment of gastrointestinal carcinomas (Hoff and Fuchs, 2003), has a more favourable tolerability profile than cisplatin and may therefore become an important alternative agent in this setting. In particular, oxaliplatin is not associated with the renal toxicity commonly seen with cisplatin. The combination of 5-FU with folinic acid and oxaliplatin has been investigated in a number of different regimens (FOLFOX, FOLFOX4, FOLFOX6, and FUFOX) in phase II clinical trials and has been shown to be effective in patients with advanced or metastatic gastric cancer, achieving overall response rates of 38–56%, median overall survival of 8.6–11.4 months, and median time to progression of 5.2–7.1 months (Louvet *et al*, 2002; Al-Batran *et al*, 2004; Chao *et al*, 2004; de Vita *et al*, 2005; Lordick *et al*, 2005). Capecitabine plus oxaliplatin either on a 3-weekly or weekly basis (XELOX and CAPOX regimens) has demonstrated similar activity to 5-FU-based combinations as first-line treatment for metastatic colorectal cancer (Borner *et al*, 2002; Scheithauer *et al*, 2003b; Zeuli *et al*, 2003; Cassidy *et al*, 2004). In addition, the combination of capecitabine and oxaliplatin is also being investigated in a phase III trial (REAL 2) in triplet combinations for the first-line treatment of gastro-oesophageal carcinoma (Sumpter *et al*, 2004; Tebbutt *et al*, 2004).

The purpose of the present pilot phase II study was to investigate the efficacy and safety of XELOX combination therapy in patients with previously untreated advanced gastric cancer.

## MATERIALS AND METHODS

### Patients

Eligible patients had histologically confirmed advanced or metastatic gastric adenocarcinoma with unidimensionally measurable disease. This was defined as at least one tumour lesion measuring ⩾1.5 × 1.5 cm^2^ with clearly defined margins on spiral CT scan, MRI, or abdominal ultrasound. For patients with proximal lesions, only those with cardia cancer were eligible, patients with GI junction tumours (AGE I) were not eligible according to the classification by Siewert *et al* (von Rahden *et al*, 2005). Patients were ⩾18 years of age with an Eastern Cooperative Oncology Group (ECOG) performance status of 0–2, and had received no prior chemotherapy for metastatic disease. Adequate haematological (absolute neutrophil count >1500/*μ*l, platelets >100 000/*μ*l), hepatic (total bilirubin <1.5 mg dl^−1^, transaminase levels <3 times the upper normal limit (UNL) or <5 times the UNL in cases of hepatic metastases), and renal (creatinine <1.5 mg dl^−1^) function was required. The protocol was approved by the institutional review board of the Korean Institute of Radiological and Medical Science, and all patients gave written informed consent before enrolment.

### Treatment schedule

Oxaliplatin 130 mg m^−2^ was administered as a 2-h intravenous infusion on the first day of each 3-week cycle. Capecitabine was administered orally at a dosage of 1000 mg m^−2^ twice daily according to the standard intermittent schedule (from the evening of day 1 until the morning of day 15 followed by a 7-day rest period).

Patients received at least two courses of XELOX unless rapid disease progression occurred after the first or second cycle. Patients who responded or who had stable disease received treatment for up to a maximum of eight cycles or until disease progression occurred.

### Dose modification for adverse events

Toxicity was graded according to the National Cancer Institute (NCI) Common Toxicity Criteria (CTC), Version 2.0 (NCI, 1999). Dose modifications for isolated abnormal haematological laboratory values were based on haematological parameters at the start of a treatment cycle. There was no scheduled sampling during a treatment cycle, so there was no scheduled collection of nadir values.

Capecitabine treatment interruption or dose reduction was not indicated for grade 1 toxicity or for events unlikely to become serious or life threatening. Treatment was interrupted in cases of grade 2 or higher events (with the exception of alopecia, nausea or vomiting, and anaemia) and was not resumed until the adverse event resolved or improved to grade 1 or 0. Capecitabine dose reduction was not required at the first occurrence of a grade 2 event. The capecitabine dose was reduced by 25% to 750 mg m^−2^ twice daily for patients who experienced a second occurrence of a given grade 2 event or any grade 3 event.

Capecitabine doses were reduced by 50% to 500 mg m^−2^ twice daily for patients who experienced a third occurrence of a given grade 2 event, a second occurrence of a given grade 3 event, or any grade 4 event. Treatment was discontinued if, despite dose reduction, a given adverse event occurred for a fourth time at grade 2, a third time at grade 3, or a second time at grade 4. If an adverse event did not improve to grade 1 or less after 3 weeks, the affected patient was withdrawn from the study.

Oxaliplatin treatment was interrupted in cases of grade 2 or higher adverse events and was not resumed until the toxicity resolved or improved to grade 1 or 0. Treatment was discontinued in cases of grade 3/4 neuropathy. If paresthesiae with pain or with persistent functional impairment were the only toxicities present at the time of the next planned administration of oxaliplatin, oxaliplatin was delayed and capecitabine continued as monotherapy. If the neurological toxicity was still present at the time of the next planned treatment cycle, oxaliplatin was discontinued permanently. In these circumstances, capecitabine was continued as monotherapy at the discretion of the investigator.

### Evaluation criteria

The primary study end point was the overall response rate as measured by the number of complete and partial responses. Secondary end points included progression-free survival (PFS), overall survival, and safety. A physical examination, including a neurological examination and complete blood counts, was performed before the first treatment cycle. Pretreatment evaluation also included biochemical analyses, chest X-ray, and CT scans to define the extent of the disease. Complete blood cell counts with differential and serum biochemistry analyses were repeated at each treatment cycle. Response was assessed radiologically every two cycles or when progression was suspected. Evaluations were performed by physical examination, chest X-ray, abdominal-pelvic CT scan, or ultrasonography. Complete response, partial response, stable disease, and progressive disease were defined according to RECIST criteria (Therasse *et al*, 2000). All the objective responses were confirmed after 4 weeks and clinical complete responses were confirmed as pathological complete responses by gastroendoscopy biopsy. CT scans were conducted to confirm the responses.

### Statistical analysis

The trial was designed using) testing procedure. Assuming a true increase in response rate of ⩾10%, 22 patients were to be included, with a target minimum response of 30% and a maximum width of 36% for the 95% confidence interval (CI). Overall response rate (with 95% CI) was calculated for all patients according to an intention-to-treat analysis. Progression-free survival was calculated from the first day of chemotherapy until the date of disease progression. Overall survival was calculated from the start of study treatment until death. Progression-free survival and overall survival curves were generated using the Kaplan–Meier method. Response duration was calculated from the date of response confirmation to the date of disease progression.

## RESULTS

### Patient characteristics

Because an ongoing phase II multicentre trial of the same regimen in an identical patient population was started before this trial was completed, only 20 of the planned 22 patients were enrolled (between December 2003 and August 2004). Baseline patient characteristics are listed in Table 1. Of the 20 patients included, 19 were evaluable for safety and tumour response. The one not evaluable patient was dropped out after two cycles of chemotherapy. The median age of the patients was 64 (range 38–75) years, and most patients (90%) had a good performance status (ECOG 1). Fifteen patients (75%) had multiple metastases involving two or more organ systems. The most common metastatic site was the liver (70%). Median follow-up duration was 11.1 months (range 8.2–14.5). Three patients (16%) had diffuse-type tumours by Lauren's classification.

### Efficacy

**Figure 1:**
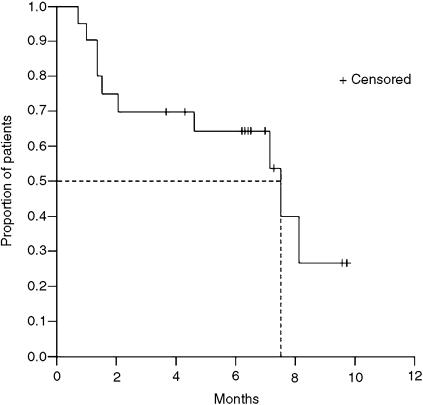
Progression-free survival (*n*=20).

The overall response rate was 65% (95% CI, 44–86%, Table 2). Two patients (10%) achieved a complete response confirmed by gastroscopic biopsy. Eleven (55%) partial responses were observed. The median duration of response in the 13 responding patients was 10.0 months (range 6.8–13.0 months). One patient had disease stabilisation, and five (25%) progressed while on treatment. The median PFS was 7.5 months (95% CI, 3.2–11.7 months) (Figure 1). The median overall survival was not reached at the time of reporting of this trial.

### Safety

A total of 97 treatment cycles (median 6, range 1–8 cycles) were administered. Haematological and nonhaematological adverse events associated with treatment are listed in Table 3. No grade 4 toxicity was observed. Grade 1 or 2 anaemia (65%) was the most common haematological event. Other grade 1 or 2 haematological events (leucopenia, neutropenia, and thrombocytopenia) were less common, and affected <40% of patients (Table 3). The only grade 3 events were leucopenia (5% of patients) and neutropenia (5% of patients).

The most common nonhaematological toxicities were neuropathy and vomiting, each of which affected ⩾60% of patients. The only grade 3 events observed were vomiting and diarrhoea, which affected 5% of patients each. These toxicities were invariably mild to moderate in severity (Table 3). Other nonhaematological toxicities were diarrhoea, hand–foot syndrome, and stomatitis.

Treatment delays or dose reductions were necessary in 30 of 97 (31%) cycles. Doses were reduced in 17 cycles (18%) as a result of neutropenia and thrombocytopenia. Treatment was delayed in 13 cycles (13%). The median dose intensities of both drugs exceeded 95%.

## DISCUSSION

Capecitabine-based combination chemotherapy for advanced gastric cancer has been shown to be active in first- and second-line treatment, achieving response rates in the range of 20–55% (Koizumi *et al*, 2003; Hong *et al*, 2004). Previous large phase III studies comparing capecitabine with bolus 5-FU plus leucovorin as first-line therapy for metastatic colorectal cancer have demonstrated superior response rates, comparable PFS and overall survival, and a favourable safety profile of capecitabine compared with 5-FU in this common gastrointestinal cancer (Twelves *et al*, 2005).

While intravenous 5-FU plus cisplatin has been widely used for the treatment of advanced gastric cancer with encouraging results, the regimen is inconvenient for patients due to the continuous 5-FU infusion and is associated with poor tolerability due to the renal toxicity and severe emesis that can occur with cisplatin. Oxaliplatin is an important chemotherapeutic agent that is being investigated in the treatment of gastrointestinal carcinomas (Hoff *et al*, 2001). It is likely to become widely used as an alternative to cisplatin if it achieves more favourable clinical outcomes. Oxaliplatin is particularly useful in advanced gastric cancer because of its good toxicity profile.

Our study shows that the XELOX regimen achieved a very good response rate and PFS in patients with advanced gastric cancer. Two patients achieved a complete response and a further 55% achieved a partial response, giving an overall response rate of 65%. The median PFS was 7.5 months (95% CI, 3.2–11.7 months). This result is particularly notable given that our patient population was relatively old with median age of 64 years. Considering that our measurable disease was defined as at least one tumour lesion measuring ⩾1.5 × 1.5 cm^2^ with clearly defined margins on spiral CT scan, MRI, or abdominal ultrasound, the objective response rate of our study could be stricter than others. According to the RECIST criteria, measurable lesions can be accurately measured in at least one dimension with the longest diameter ⩾20 mm using conventional techniques or ⩾10 mm with spiral CT scan. While the usual limitations of cross-study comparisons should be taken into account, these findings compare favourably with two recent studies investigating the efficacy of cisplatin in combination with capecitabine as first-line therapy in patients with advanced gastric cancer, which reported overall response rates of 46 and 55%, respectively (Kim *et al*, 2002; Jin *et al*, 2005). Median time to progression reported in one study was 6.3 months (Kim *et al*, 2002).

XELOX also had a good safety profile. No grade 4 toxicity was reported and grade 3 haematological and nonhaematological events were rare (<5% of cycles/patients). The most common toxicities reported were vomiting, neuropathy, and diarrhoea, which were generally of mild to moderate intensity. The toxicity profile reported with XELOX in our trial compares favourably with that of cisplatin–capecitabine as reported by Kim *et al* (2002), who observed grade 3/4 neutropenia in 33% of patients (*vs* 5% of patients in the present trial). The rate of grade 2 peripheral neuropathy in our study may be considered somewhat low. In a study of oesophageal cancer, Mauer *et al* (2005) reported a rate of 26% for grade 2/3 peripheral neuropathy.

A large ongoing phase III trial (REAL 2) is currently investigating replacing 5-FU with capecitabine and cisplatin with oxaliplatin in triplet combinations with epirubicin for the first-line treatment of gastro-oesophageal carcinoma (Sumpter *et al*, 2004; Tebbutt *et al*, 2004). Early indications from the REAL 2 study indicate that the combination of capecitabine and cisplatin could be effective in triplet therapy. A planned interim analysis showed that capecitabine at a dose of 1000 mg m^−2^ day^−1^ had a better toxicity profile than 5-FU: the rate of grade 3/4 diarrhoea, stomatitis, or hand–foot syndrome was 5% in capecitabine-treated patients compared with 17% in 5-FU-treated patients (Tebbutt *et al*, 2004). In a second planned interim analysis of a higher capecitabine dose of 1250 mg m^−2^ day^−1^, complete or partial responses were seen in 31% of patients receiving epirubicin/cisplatin/5-FU compared with 48% for those receiving epirubicin/oxaliplatin/capecitabine (Sumpter *et al*, 2004). The rate of grade 3/4 fluoropyrimidine-related toxicity was 11% in capecitabine-treated patients (1250 mg m^−2^ day^−1^) compared with 13% in 5-FU-treated patients.

In summary, XELOX combination chemotherapy is highly active in patients with previously untreated advanced gastric cancer. This novel combination regimen overcomes issues of poor tolerability and inconvenience associated with other regimens currently used in this cancer type. On the basis of these promising results, we have initiated a large phase II multicentre study of XELOX in advanced gastric cancer. A phase III adjuvant trial is also planned.

## Figures and Tables

**Table 1:** Patient characteristics (*n*=20)

**Characteristic**	**Number of patients (%)**
*Age (years)*	
Median	64
Range	38–75
	
*Sex*	
Male	17 (85)
Female	3 (15)
	
*ECOG performance status*	
1	18 (90)
2	2 (10)
	
*Metastatic site* a	
Liver	14 (70)
Lung	2 (10)
Abdominal lymph node	11 (55)
Soft tissue	1 (5)
	
*Number of metastatic sites*	
1	5 (25)
2	9 (45)
⩾3	6 (30)

a Patients could have more than one metastatic site.

**Table 2:** Response to treatment (*n*=20)

**Response**	**No. of patients (%)**
Confirmed response	13 (65)
Complete response	2 (10)
Partial response	11 (55)
Stable disease	1 (5)
Progressive disease	5 (25)
Not assessable	1 (5)

**Table 3:** Haematological and nonhaematological adverse events (*n*=20)

	**Grade (% of patients)**			
	**1**	**2**	**3**	**4**
Anaemia	60	5	0	0
Leukopenia	30	10	5	0
Neutropenia	20	10	5	0
Thrombocytopenia	10	15	0	0
Vomiting	45	15	5	0
Stomatitis	10	5	0	0
Diarrhoea	15	10	5	0
Hand–foot syndrome	15	5	0	—
Neuropathy	55	5	0	0
